# Understanding mental health challenges in health students

**DOI:** 10.1186/s12909-026-09836-x

**Published:** 2026-07-02

**Authors:** Sergio Baldassin, Anderson Sousa Martins-Da-Silva, João Mauricio Castaldelli-Maia, Nilson Silva

**Affiliations:** 1 Department of Neuroscience, ABC Health University Center, Santo André, Brazil; 2 Support Center of the Hospital Estadual Mário Covas, Santo André, Brazil; 3 Department of Psychiatry, Medical School, University of São Paulo, São Paulo, Brazil; 4 Department of Psychiatry, Medical School, University of Santo Amaro, São Paulo, Brazil; 5 Department of Neuroscience, FMABC Medical School of Saúde ABC University, Av. Lauro Gomes, 2000 - Vila Sacadura Cabral, Santo André - SP, 09060-870 Brazil

**Keywords:** Health personnel, Depression, Anxiety, Mental health

## Abstract

**Background:**

Health challenges among healthcare students, particularly those in Medicine, Nursing, and Occupational Therapy, have garnered increasing attention. This study aimed to assess the prevalence of depression and anxiety symptoms among 780 undergraduate students and explore associated factors and help-seeking behaviors.

**Methods:**

Data from 780 medical records were retrospectively analyzed. In addition to a complete standardized anamnesis, students were invited to complete the Beck Depression Inventory and the Spielberger Anxiety Inventory.

**Results:**

Out of 11,567 consultations conducted over 21 years (which generated 780 medical records), 584 students completed both the BDI and STAI-T questionnaires and were included in the final analysis. Medicine accounted for 337 students (57.7%), Nursing for 134 students (22.9%), and Occupational Therapy for 114 students (19.5%). Participants’ ages ranged from 17 to 58 years.

Medical students had lower mean ages and fewer severe depression cases, while nursing students showed higher rates of divorced, widowed, brown, and black individuals. Occupational therapy students had more severe depression symptoms and higher averages in the affective cluster of the Beck Inventory for Depression. Medical students exhibited higher levels of spontaneous help-seeking, while nursing and occupational therapy students were more likely to be referred for help by professors. Significant associations were found between the graduation and variables such as family mental health treatment, referral sources, and indications for psychotherapy.

The severity of depression symptoms significantly influenced help-seeking behaviors, with students experiencing non-severe depression being more inclined to seek help spontaneously.

**Conclusions:**

These findings underscore the importance of proactive mental health support and tailored interventions within healthcare education, ultimately contributing to the mental health, self-care, and skill development of students, teachers, and institutions.

**Trial registration:**

Approved by the Research Ethics Committee in Certificate of Presentation of Ethical Assessment opinion 31210620.0.0000.0082, number: 4.098.082. The research protocol was approved on August 16th, 2000. Clinical trial number: not applicable.

## Background

The education of students in the health sector demands grit, tenacity, and resilience [1] in the face of challenges during their undergraduate studies and training [2].

However, mental health issues such as depression, anxiety, burnout, and suicide [3] are pressing concerns in higher education, particularly among students in healthcare-related fields of study.

Research suggests that the demanding nature, especially of medical training, combined with academic stressors such as limited time for leisure and personal care, various forms of harassment, and years of clinical exposure to several types of suffering, may predispose students to challenges in maintaining their physical and emotional health. Recognizing the incidence and addressing the factors associated with depression and anxiety during the formative period becomes essential for implementing effective support systems and promoting student well-being.

Recent discussions [4] intensified by the SARS-CoV-2 pandemic, have begun to acknowledge the significance of emotional well-being in student training. It’s crucial to understand that promoting students’ health requires a collaborative effort between institutions and students themselves, including mental health [5].

Despite progress, some professors, often excellent physicians with no formal training in medical education, remain unaware of the pedagogical importance of socio-emotional balance and its impact on patient care and healthcare costs. Neglecting self-care not only affects professional performance but also compromises patient safety, with consequences in public and private health costs [6–8].

This study presents the results collected over 21 years, including thousands of consultations with semi-structured and well-documented medical records. Understanding these data and implementing coping mechanisms are vital for ensuring the well-being of both students and future healthcare professionals. Often, those who neglect their care serve poorly, and if they are consistently tired or moody, they can lose sight of the essence of their profession, which is to care for others, since there is no health without mental health [2, 9].

## Methods

This is an exploratory, retrospective, initially descriptive study, based on data collected from 1998 to 2019 in medical records, on the first consultation, of care at a university support service for students in the health area. This was a convenience sample; we included graduations with more than 100 consultations.

The staff of our service comprises psychiatrists and psychologists. Informed consent to participate was obtained from all of the participants. Approved by the Research Ethics Committee in CAEE opinion 31210620.0.0000.0082, number: 4.098.082. Each medical record collects 37 pieces of information: type of care (urgent or common consultation), place of birth, date of birth, referrer, address, residence, grade attended, years of preparatory course for college admission, marital status, whether they live with the family, gender, religion, self-reported color, whether they were medicated, whether psychotherapy was indicated, whether they referred to a mental disorder in the family, whether they had previously undergone treatment, what was the main reason for seeking it, what was the age at which they decided their career in health, if they already had a family member who was a health professional, if there was any traumatic event suffered in the graduation, if any family member was undergoing mental treatment, and what was the degree of stress reported in the first contact with the cadaver.

In addition to a complete standardized anamnesis, students were invited to complete the Beck Depression Inventory [10] (BDI)and the Spielberger Anxiety Inventory [11] (STAI-T), using the following cut-off points: depressive symptoms have been considered for BDI: 0–9 - minimum or absent, 10–17 - mild, 18–29 - moderate, and 30–63 - severe depression. The scoring for the STAI-S has been based on the following cut-offs: <33 - mild, 33–49 - moderate,>49 – high anxiety.

The spreadsheet created from the beginning for this academic service automatically divided and recorded the BDI into: 1] cognitive cluster summing the items: Worthlessness (B3 + B7 + B14), Guilt (B5 + B6 + B8), Excessive worry (B20), Indecision (B13) and Suicide (B9), 2] somatic cluster gathering the items: Tiredness (B15 + B17), Sleep (B16), Appetite (B18 + B19) and Sexuality (B21), and 3] affective cluster gathering the items: Sadness (B1 + B10), Anhedonia (B4 + B12) and Irritability (B11).

The existence of associations between two categorical variables was verified using the Chi-square test, or in cases of small samples, by Fisher’s exact test. When there was an association, the standardized adjusted residual was used to identify local differences – cells with absolute values above 1.96 indicated evidence of (local) associations between the categories related to these boxes. The internal consistency of the BDI scales (total and domains) and STAI was evaluated using Cronbach’s Alpha.

The comparison of means between more than two groups was performed via ANOVA. The normality of the data distribution was verified using the Kolmogorov-Smirnov test. In case of violation of normality in the distribution of data, the means were compared using the non-parametric Kruskal-Wallis test. Once the differences in means were detected in the Kruskal-Wallis test, the localization of the differences was performed using multiple Dunn-Bonferroni comparisons, maintaining a global significance level of 5%.

Subsequently, to evaluate the mean behavior of the BDI in its affective cluster among the types of students in the health area (Medicine, Nursing, and Occupational Therapy), adjusted for demographic and clinical characteristics, simple (univariate) and multiple (multivariate) linear regressions were employed. In the initial multivariate model, all explanatory variables were included, and then non-significant variables at 5% were excluded one by one in order of significance. Linear regression assumes normality in the distribution of the dependent variable, which was verified using the Kolmogorov-Smirnov test. A significance level of 5% was adopted for all statistical tests. Statistical analyses were performed using the statistical software SPSS 20.0 and STATA 12.

## Results

Out of 11,567 consultations conducted over 21 years (which generated 780 medical records), 584 students completed both the BDI and STAI-T questionnaires and were included in the final analysis. Medicine accounted for 337 students (57.7%), Nursing for 134 students (22.9%), and Occupational Therapy for 114 students (19.5%). Participants’ ages ranged from 17 to 58 years, with a mean age of 23.4 years (SD = 5.8 years).

Medical students had the highest percentage of male students (33.8%), were single (99.1%), white (84.3%), and of the yellow race (6.7%), identified as Catholic (48.3%), and reported no affiliation with a religion (31.3%), and sought the service spontaneously (63.9%). And they were the only ones referred to our service by the family (1.2%), the administration (0.3%), and the board of directors (0.6%).

Nursing students had the highest percentages of being married (14.2%), divorced (7.5%), and brown (31.8%), as well as living with their families (93.3%), and were predominantly first-year students (57.5%). It is the second-highest percentage of male students (13.4%).

Occupational Therapy students had the highest percentage of self-reported mental treatment in the family (88.6%) and the second highest percentage of white students (72.0%).

Students in Nursing and Occupational Therapy graduations had the highest percentage of referrals to the support service by professors (27.8% and 25.4%) and indications for psychotherapy after the first consultation (95.9% and 84.3%).

And there were students in the 1st year: 231 (39.6), in the 2nd year: 143 (24.5), in the 3rd year: 102 (17.5), in the 4th year: 64 (11), in the 5th year: 26 (4.5) and in the 6th year: 18 (3.1).

### Diagnostics

The main diagnoses, based on the International Classification of Diseases (ICD-10), were distributed as follows: Depressive disorders: 295 (50.4%), Anxiety disorders: 267 (45.6%), Eating disorders: 8 (1.4%), Personality disorders: 4 (0.7%), Developmental disorders: 2 (0.3%), and other disorders (including behavioral, conduct, social functioning, hyperkinetic, impulsive, intelligence, organic brain, and substance use disorders): 1 (0.2%) each.

### Depressive symptoms levels

Significantly (*p* = 0.025), 19.2% were in the range (BDI) of minimal or no depression (0 to 9 points), 34.8% in the mild range, 34.8% in the moderate range, and 11.2% in the severe depression range. Of these, 20 (21.1%) were from Occupational Therapy, 14 (11.2%) from Nursing, and 24 (8.0%) from Medicine.

### Features by graduation

The association between graduation and the following variables was verified: mental treatment in the family (*p* = 0.014), referral source (*p* = 0.002), and indication of psychotherapy (*p* < 0.001).

A significant association was observed between the classification of BDI severity ranges and graduation (*p* = 0.025). Thus, Occupational Therapy students had a higher percentage of BDI in the severe range (21.1%) compared to Nursing (11.2%) and Medical (8.0%) students.

**Fig. 1:**
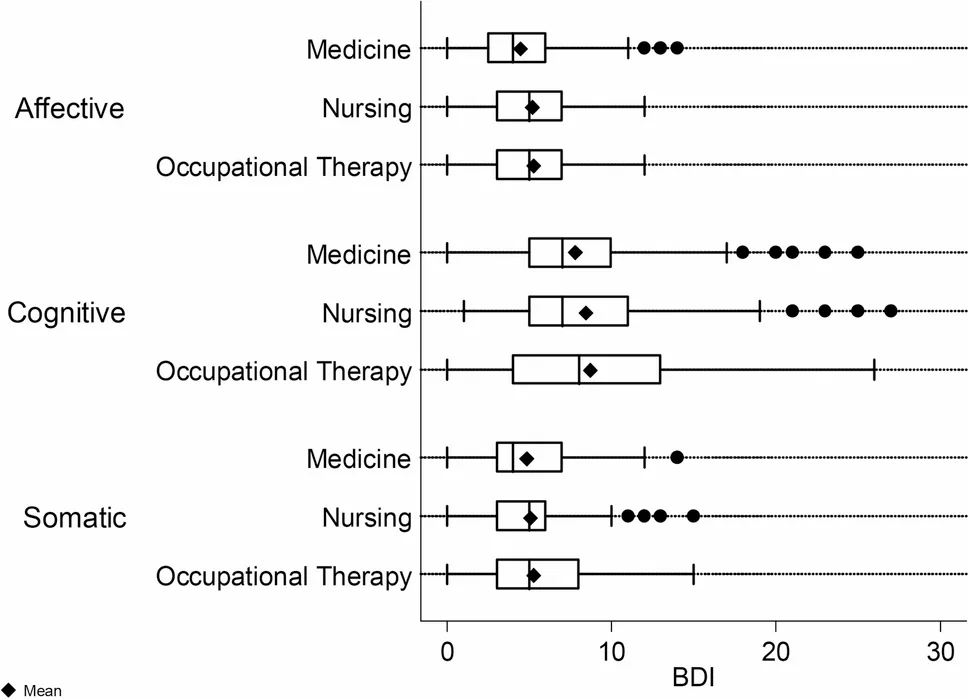
Boxplot of domains of the BDI clusters by graduation

The BDI showed good internal consistency, with Cronbach’s Alpha = 0.871. And significantly (*p* = 0.027), the analysis of variance revealed differences only in the means of the affective cluster (Table 1; Fig. 1) of the BDI between the cgraduations. Thus, medical students (4.5 points) had a lower mean than nursing (5.1 points) and occupational therapy (5.3 points). There were no differences in the means of the nursing students’ group with those of the other groups.

**Table 1:** BDI summary measures (total and clusters) and STAI by graduation

	Mean	Standard Deviation	Minimum	Maximum	1° Quartile	Median	3° Quartile	*p*
BDI	17,5	9,2	0,0	52,0	11,0	17,0	23,0	0,296
Medicine	16,8	8,5	1,0	48,0	11,0	17,0	22,0	
Nursing	18,1	9,5	2,0	52,0	11,0	17,0	24,0	
Occupational Therapy	19,0	10,3	0P,0	42,0	11,0	16,0	28,0	
BDI-Affective	4,8	2,8	0,0	14,0	3,0	5,0	6,0	0,027
Medicine	4,5^A^	2,7	0,0	14,0	2,3	4,0	6,0	
Nursing	5,1	2,9	0,0	12,0	3,0	5,0	7,0	
Occupational Therapy	5,3^B^	2,8	0,0	12,0	3,0	5,0	7,3	
BDI-Cognitive	8,1	4,9	0,0	27,0	5,0	7,0	11,0	0,613
Medicine	7,8	4,5	0,0	25,0	4,8	7,0	10,0	
Nursing	8,4	5,1	1,0	27,0	5,0	7,0	11,0	
Occupational Therapy	8,7	5,7	0,0	26,0	4,0	8,0	13,0	
BDI-Somatic	5,0	3,1	0,0	15,0	3,0	5,0	7,0	0,418
Medicine	4,8	3,0	0,0	14,0	3,0	4,0	7,0	
Nursing	5,0	3,2	0,0	15,0	3,0	5,0	6,3	
Occupational Therapy	5,3	3,4	0,0	15,0	2,8	5,0	8,0	
STAI	45,2	5,7	26,0	64,0	41,0	45,0	49,0	0,628^a^
Medicine	45,3	5,5	30,0	64,0	42,0	45,0	49,0	
Nursing	45,2	6,1	26,0	61,0	42,0	45,0	49,0	
Occupational Therapy	44,7	6,0	27,0	59,0	41,0	45,0	48,0	

p - Descriptive level of the Kruskal-Wallis test or ANOVA ^(a)^

(A) and (B) present different means according to Dunn-Bonferroni comparisons

Subsequently, simple and multiple linear regressions were adjusted, with BDI as the dependent variable and gender, age, marital status, color, religion, living with the family, year of graduation, mental treatment in the family, professional who performed the referral, and indication of psychotherapy characteristics that were different by graduation. Graduation (*p* = 0.015), marital status (*p* = 0.017), and mental treatment reported in the family (*p* = 0.026) remained significant.

**Fig. 2:**
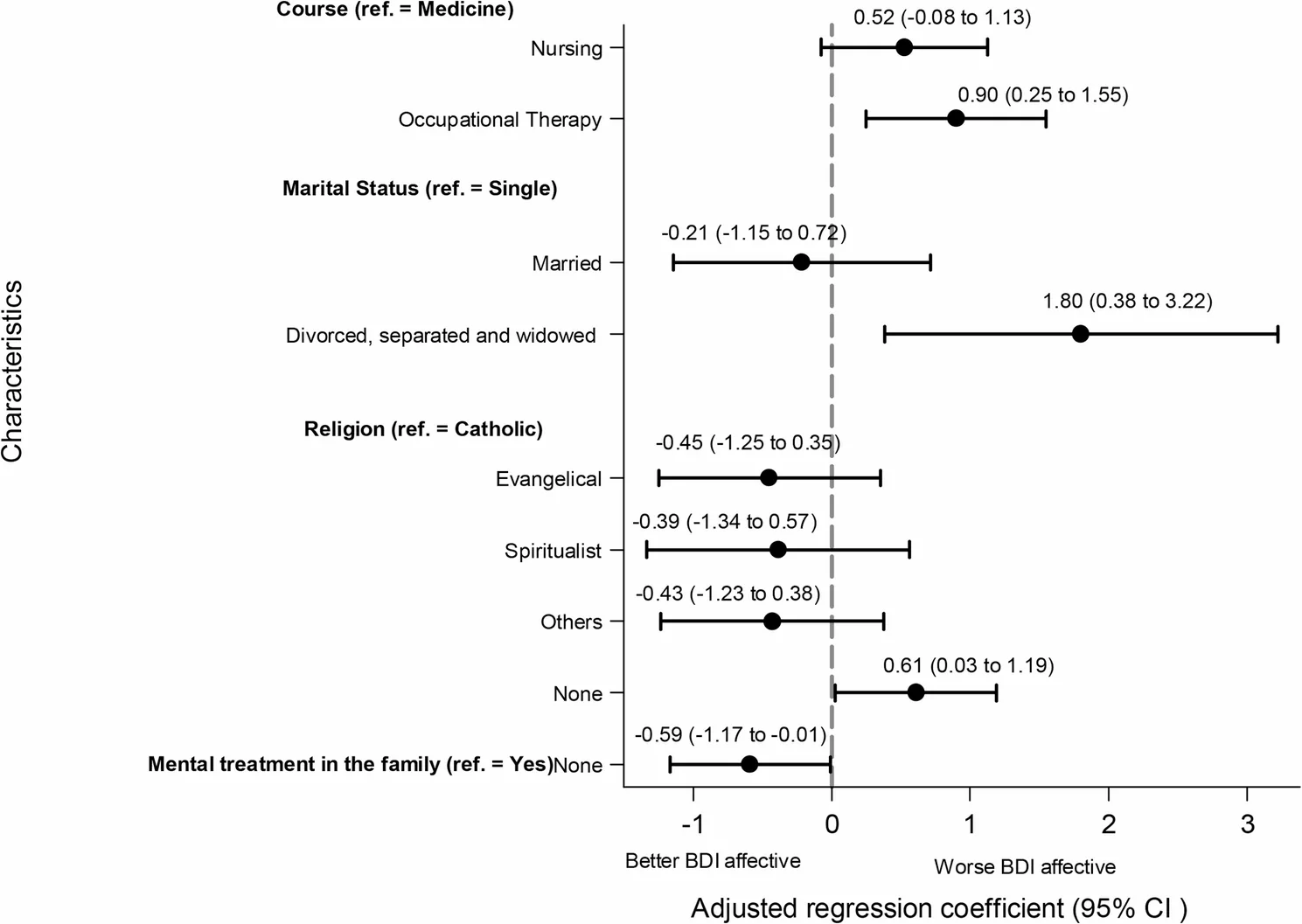
Estimates of final regression coefficients for BDI affective cluster

In addition, multiple linear regression models were adjusted to evaluate the effect of graduation on affective BDI, adjusted for gender, age, marital status, color, religion, living with the family, year of graduation, mental treatment in the family, professional who performed the referral, and indication of psychotherapy. After adjusting the initial multivariate model, the non-significant variables at 5% were excluded one by one in order of significance. As shown in Table 2; Fig. 2, the following variables remained significant: graduation (*p* = 0.019), marital status (*p* = 0.035), religion (*p* = 0.048), and mental treatment in the family (*p* = 0.048).

**Table 2:** Results of initial and final multiple linear regression models for BDI – affective cluster

	Multivariate Model			
	Initial		Final	
	Row Coefficient (CI95%)	*p*	Adjusted Coefficient (CI95%)	*p*
Grupo (ref. = Medicine)		0,122		0,019
Nursing	0,45 (-0,29 a 1,19)	0,232	0,52 (-0,08 a 1,13)	0,088
Occupational Therapy	0,81 (0,03 a 1,59)	0,043	0,90 (0,25 a 1,55)	0,007
Male Gender (ref. = Female)	-0,15 (-0,81 a 0,52)	0,667	-	-
Age (years)	0,00 (-0,05 a 0,06)	0,939	-	-
Marital status (ref. = Single)		0,005		0,035
Married	-0,31 (-1,51 a 0,89)	0,616	-0,21 (-1,15 a 0,72)	0,653
Divorced/Separated/Widowed	2,59 (0,78 a 4,40)	0,005	1,80 (0,38 a 3,22)	0,013
Skin color (ref. = White)		0,993		
Brow	-0,10 (-0,85 a 0,65)	0,789	-	-
Black	0,04 (-1,63 a 1,71)	0,964	-	-
Yellow	-0,08 (-1,30 a 1,14)	0,896	-	-
Religion (ref. = Catholic)		0,051		0,048
Evangelical	-0,32 (-1,20 a 0,57)	0,481	-0,45 (-1,25 a 0,35)	0,272
Spiritualist	-0,37 (-1,43 a 0,68)	0,487	-0,39 (-1,34 a 0,57)	0,426
Others	-0,40 (-1,28 a 0,48)	0,374	-0,43 (-1,23 a 0,38)	0,296
None	0,75 (0,09 a 1,41)	0,026	0,61 (0,03 a 1,19)	0,041
Living with the family - no (ref. = Yes)	0,06 (-0,6 a 0,72)	0,853	-	-
Year of study (ref.=1st)		0,194		
2nd	-0,09 (-0,77 a 0,59)	0,802	-	-
3rd	0,58 (-0,16 a 1,32)	0,125	-	-
4th	-0,73 (-1,65 a 0,20)	0,124	-	-
5th	0,10 (-1,22 a 1,41)	0,886	-	-
6th	0,62 (-1,01 a 2,26)	0,454	-	-
Mental treatment referred in family - No (ref. = Yes)	-0,58 (-1,23 a 0,06)	0,078	-0,59 (-1,17 a -0,01)	0,048
Referred by (ref.= Spontaneous)		0,494		
Colleagues	0,50 (-0,16 a 1,16)	0,140	-	-
Professors	-0,34 (-1,04 a 0,37)	0,344	-	-
Family	1,38 (-2,57 a 5,34)	0,492	-	-
Administrative board	-0,92 (-6,56 a 4,71)	0,747	-	-
Doctors	1,11 (-1,21 a 3,43)	0,350	-	-
Medical School Board	-1,83 (-5,80 a 2,14)	0,366	-	-
Others	0,68 (-2,58 a 3,94)	0,682	-	-
Psychotherapy Indicated - No (ref. = Yes)	-0,16 (-1,07 a 0,76)	0,733	-	-
R^2^	0,0885		0,0501	
R^2^ adjusted	0,0312		0,0339	
Descriptive level of the Kolmogorov Test - Smirnov	0,108		0,074	

Thus, adjusted for the characteristics present in the final model, Occupational Therapy students have, on average, a higher affective BDI score (0.90 points) compared to medical students, with no differences in means between nursing and medical students (*p* = 0.088). Regarding the other characteristics, divorced, separated, or widowed students have a higher mean score than single students (on average, 1.80 points higher), and students with no religion have a mean score 0.61 points higher than catholics. On the other hand, those without mental treatment in the family have a lower score than students with this condition (0.59 points less, on average).

## Discussion

Medical students, often young and living independently for the first time( above 90% of the other graduates lived with their parents), had the lowest average age and were more likely to seek help spontaneously (63.9%) than Nursing (54.1%) and Occupational Therapy (51.8%) students, possibly due to early exposure to the service, reinforcing the need for awareness of this service throughout the whole university. Regardless of whether they were referred or sought our service spontaneously, our sample consisted only of students who sought help within the university. This help-seeking pattern underscores the importance of accessible support services for mild and moderate cases for all health graduations [12]. Moreover, unlike other graduations, medical students have a significant demand for help (*p* < 0.001), even in their last years of graduation.

The study highlights the necessity of educating medical professors about guiding students to seek help, given their lower referral rates (13.1%) compared to nursing (27.8%) and occupational therapy (25.4%) professors. This prompts questions about the perception of self-care’s importance among medical educators. Based on some of these findings, some medical school faculty may perpetuate the notion that self-care is secondary or unnecessary [13]. Another possible explanation for these lower referral rates from medical professors might be the high rate of spontaneous help-seeking (63.9%) from these students.

The study also observed variations in psychotherapeutic treatment recommendations, with nursing and occupational therapy students receiving more referrals than medical students, with the latter being more likely to receive psychotropics. This difference may indicate a greater receptiveness to non-pharmacological approaches or varying levels of mental health issue severity [13, 14].

The analysis revealed no significant differences in anxiety disorders across graduations (*p* = 0.259), with moderate anxiety prevailing in 77.4% of cases. This suggests that anxiety is prevalent among students and healthcare professionals, potentially leading to its trivialization and inadequate management [15].

Depression and anxiety disorders were the most frequent diagnoses, which are potential risk factors for ideas of death, suicide attempts, and suicide in health professionals later on [16].

While there was no significant difference in the cognitive cluster of BDI (Table 1; Fig. 1) among Graduations, the highest mean values were predominant. This cluster includes feelings of hopelessness, worthlessness, guilt, excessive worry, indecision, and suicide ideation. Given the high demands placed on students in the health field, the elevated scores in this cluster were somewhat expected, as observed in previous studies [13, 14].

To improve the quality of life of health students, many institutions are already seeking to create support services and make structural changes in the way students are assessed [17, 18], adopting online support services [19, 20], assessments for new students [21], and physical care services. And accreditation institutes, like the Brazilian Federal Council system, also score schools that offer psychological, pedagogical, mentoring, and medical support 22, 23. Despite these advances, few student support services produce and share knowledge [7, 22, 24, 25].

An intriguing finding is that there was a significant association between graduation and the presence of psychiatric treatment reported in members of the student’s family (p = 0.014). Thus, 88.6% of the students in the occupational therapy graduation reported having a family member with a history of previous psychiatric treatment, compared to 75.9% of the nursing students and 76.2% of the medical students. These high prevalences should be considered in the context of reflected shame (worries for their/others’ reputation because of others’/their mental health problems), since healthcare students demonstrated a high level of this shame component [26]. Shame towards mental health is often a barrier to seeking help or revealing their mental health issues to others, making their mental health problems worse and increasingly difficult to manage [26].

Moreover, the research underscores the high occurrence of depressive and anxiety disorders among healthcare students, emphasizing the urgency of effective interventions and support systems. Implementing initiatives like outpatient clinics and comprehensive mental health services is essential for enhancing student well-being and cultivating competent and responsible future healthcare professionals [27].

### Limitations

The data of this study are the result of a limited number of consultations, mostly with medical students. And they were found only in a survey of one medical school, which may not be representative of all schools in the country or of the respective graduations.

Our sample includes only help-seeking students, which introduces selection bias. At the same time, some students might be receiving treatment elsewhere and weren’t included in our sample.

## Conclusion

This study underscores the prevalence of depression and anxiety among healthcare students and the importance of tailored support mechanisms. Findings suggest the need for proactive mental health initiatives within healthcare education, emphasizing early intervention and destigmatization of help-seeking behaviors. Further research is warranted to explore longitudinal trends and evaluate the efficacy of intervention strategies.

## Data Availability

All data generated or analysed during this study are included in this published article.

## Abbreviations

BDI: Beck Depression Inventory
ICD-10: International Classification of Diseases – 10th revision (ICD-10)
STAI-T: Spielberger Anxiety Inventory
SPSS: Statistical Package for the Social Sciences
